# Crafting Task and Cognitive Job Boundaries to Enhance Self-Determination, Impact, Meaning and Competence at Work

**DOI:** 10.3390/bs9120136

**Published:** 2019-12-03

**Authors:** Severin Hornung

**Affiliations:** Institute of Psychology, University of Innsbruck, Innrain 52, A-6020 Innsbruck, Austria; severin.hornung@uibk.ac.at

**Keywords:** job crafting, proactive work behavior, growth need strength, intellectual stimulation, situational constraints, psychological empowerment, survey research, structural equation modeling

## Abstract

Job crafting refers to physical and cognitive changes in task or relational work boundaries, enacted by individuals to recreate their work experience in a more motivating and rewarding way, and to realize self-actualization, growth, and meaning at work. This study tests a model of individual, interpersonal, and organizational antecedents and motivational outcomes of situation-directed task and self-directed cognitive job crafting. Employee survey data (N = 1196) from a Chinese telecommunications company permitted confirmatory factor analysis and structural equation modeling. Antecedents were each measured with three-item versions of established scales, a two-dimensional scale on task and cognitive job crafting was newly developed, and a four-dimensional model of psychological empowerment captured motivational effects. Structural modeling confirmed a partial mediating role of job crafting between antecedents and empowerment. Individual growth requires strength and intellectual stimulation from one’s leader that is positively related to both tasks and cognitive crafting, while exposure to organizational constraints triggered task crafting only. Confirming differential motivational effects, task crafting predicted control-oriented empowerment dimensions of self-determination and impact, whereas cognitive crafting affected person-oriented dimensions of meaning and competence. Interpreted as a micro-emancipatory form of self-management, job crafting offers some new insights into leadership, coping, work design, work orientations, and motivation. Practical and research implications of this are discussed.

## 1. Introduction

Job crafting is currently one of the most highly discussed topics in work and organizational psychology [1,2,3]. Exemplifying proactive behavior, job crafting refers to “the physical and cognitive changes individuals make in the task or relational boundaries of their work” [1] (p. 179). Through such actions, employees develop their occupational identities and job design, making their work more personally meaningful, motivating, and satisfactory. While many studies have covered this topic, few reflected the original conceptualization of job crafting. The research was either conceptual or used qualitative methods [1,2,4], while quantitative studies were constrained by lacking survey measures [5]. This changed with the development of a job crafting scale based on the job demands-resources model [6,7,8]. This scale [6] has been widely used to examine antecedents (e.g., individual differences) and outcomes (e.g., work engagement, performance) of job crafting aimed at improving work characteristics by increasing job resources (e.g., task autonomy) and challenge demands (e.g., task complexity) and reducing stressful hindrance demands (e.g., role conflicts) [3]. Important insights notwithstanding, the theoretical foundation and operationalization of job crafting according to the job demands-resources model [7,8] is somewhat detached from the initial conceptualization [1,2,8]. Alternative scales have been suggested, but these either do not include the distinction between (situation-directed) task and (self-directed) cognitive crafting, or suffer from psychometric shortcomings [9,10]. Task and cognitive crafting resemble two directions of proactive behavior, which can be aimed at affecting changes in the work environment (e.g., job content or working conditions) or at changing oneself (e.g., own thoughts or behavior) to improve alignment between the job and personal attributes [11]. Task crafting describes modifications of the number, scope, or type of job duties, for example, taking on new tasks, changing work processes, or devoting extra time to certain aspects of the job [2]. Cognitive crafting involves redefining or reframing one’s occupational role, tasks, and job boundaries, for example, viewing the work in a larger context or focusing on personally meaningful aspects (e.g., broader benefits for oneself and others) [1]. Task and cognitive crafting complement each other as situation-directed and self-directed forms of control [12]. To test a model of antecedents and outcomes of task and cognitive crafting, a scale to measure these two dimensions, which were not fully represented in prior research, was developed. While both task and cognitive crafting can affect social relationships at work (e.g., collaborative tasks, attitudes towards customers), relational crafting of social interactions was not included as a separate dimension [8]. With specified cognitive and task crafting as the focal constructs, their individual, interpersonal, and organizational antecedents and psychological outcomes will be discussed next.

Similar to other forms of proactive behavior, the propensity to craft one’s job is influenced by individual differences [3,11,13,14]. Early research on proactivity has shown that organizational newcomers with a high need for control engage in active socialization tactics of positive framing, feedback-seeking, relationship building, and job change negotiation [15]. Later, a proactive personality, a disposition towards exercising influence and initiating change, and learning goal orientation, and the tendency to seek out opportunities for mastery experiences have been shown to predict proactive behaviors [11]. The present study focuses on individual growth strength needs, the desire to obtain satisfaction from achievement, mastery, and skill development at work, which is related to the need for control, a proactive personality, and learning orientation [16,17,18]. Proposing that growth-oriented individuals are particularly active in shaping and redefining their jobs, Hypothesis 1 predicts that the need for growth and strength relates positively to both task crafting (H1a) and cognitive crafting (H1b).

The proposition that supportive leaders facilitate job crafting is intuitively plausible, however, the empirical results are less clear-cut [5,19]. To reexamine previous findings, the present study draws on transformational leadership literature. Transformational leaders influence the values, attitudes, and beliefs of their followers, making them feel motivated and empowered to perform above and beyond their job duties. Transformational leadership is multi-dimensional, including articulating a vision, inspirational motivation, individual consideration, and intellectual stimulation [20,21]. The last dimension is the most relevant here. Intellectual stimulation refers to leader behavior that encourages employees to challenge their assumptions about their work and come up with new ideas on how to perform tasks in a better way. Thus, intellectual stimulation is aimed at positively influencing behavioral and thinking patterns of employees, corresponding with notions of task and cognitive crafting. Hypothesis 2 formalizes the assumption that intellectual stimulation by one’s leader will relate positively towards both task crafting (H2a) and cognitive crafting (H2b).

The impetus to engage in job crafting does not necessarily need to be positive. Workers use job crafting to overcome obstacles, reframe unfavorable conditions, or change situations they are dissatisfied with [2,5,6]. The concept of situational constraints [22] captures organizational conditions that hinder the attainment of work goals (e.g., dysfunctional rules and regulations), triggering negative employee responses, such as frustration, counterproductive behaviors, and impaired well-being [23]. However, a central insight of the coping literature is that individuals can respond to stressful demands in different ways. Positively reframing potentially stressful situations as challenges rather than as threats and taking problem-focused actions to constructively deal with or overcome stressors are considered to be effective [24,25]. Cognitive crafting and task crafting reflect these active coping strategies [2,3,5]. Accordingly, Hypothesis 3 postulates that situational constraints will relate positively to task crafting (H3a) and cognitive crafting (H3b).

Effects of job crafting were operationalized with the concept of psychological empowerment, defined as a comprehensive motivational construct, reflecting an individual’s work role orientation in terms of cognitions of self-determination, impact, meaning, and competence [26,27,28]. Assuming that workers engage in job crafting to make their jobs more personally motivating, meaningful, and satisfactory, self-initiated changes in task and cognitive boundaries should generate a sense of empowerment [1,5,29]. However, task and cognitive crafting likely relate differently to the four dimensions of empowerment, which have both shared and distinctive features [27]. Self-determination and impact are control-focused, referring to autonomy and influence at work [30]. Self-determination describes the degree of authority and discretion in fulfilling job tasks, including decisions on work goals, methods, and timing [31]. Impact refers the broader work environment, such as management decision-making concerning the work unit or department. In contrast, meaning and competence are more self-focused, referring to evaluations of alignment between the job and one’s own values and skill sets [32]. Meaning is the subjective importance of one’s work, based on correspondence with their personal values and ideals [29]. Competence is defined as self-efficacy beliefs regarding the ability to perform one’s work role. Task crafting is outward-directed or situation-focused, aimed at changing conditions of one’s work by exercising control and influence. Cognitive crafting is inward-directed or self-focused, involving changing own thoughts and beliefs about one’s job and work identity. Thus, task crafting should be more relevant for control-oriented empowerment dimensions, while cognitive crafting is more proximal to the self-oriented components. Hypothesis 4 reflects these considerations, predicting that task crafting will relate positively to self-determination (H4a) and impact (H4b), whereas cognitive crafting will relate to meaning (H4c) and competence (H4d).

## 2. Materials and Methods

Part of a larger research, consulting, and outreach collaboration between the business school of a university based in Hong Kong and a large telecommunication company in mainland China, survey data were gathered through an online electronic questionnaire. Out of more than 20,000 employees, a stratified sample of 1500 was selected by the HR department as a cross section of functions (i.e., technical, administrative, and service staff) and ranks (i.e., rank-and-file workers, line supervisors, and managers). Invitations were sent via the internal email system along with letters of informed consent and access codes to an internal website where the questionnaire was filled out.

### 2.1. Sample

Out of the 1500 invited employees, N = 1196 participated, a 79.7% response rate; 554 were men (46.3%) and 642 women (53.7%); the mean age and organizational tenure were 32.78 (SD = 6.83) and 7.08 (SD = 4.33) years, respectively; education ranged from middle school (11; 0.9%), high school (139; 11.6%), associate degree (507; 42.4%), bachelor’s degree (499; 41.7%), to a master’s degree (40; 3.3%).

### 2.2. Measures

All measures were based on self-reports using a 7-point scale (1 = “strongly disagree”; 7 = “strongly agree”) and were administered in Chinese, following an iterative process of back-and-forth translation, discussion, and consensual decisions of several bilingual researchers. The job crafting measure was developed for this study as existing alternatives were suboptimal for the purpose. Out of five initial items per dimension, four items each for task (α = 0.76) and cognitive crafting (α= 0.81) were retained, based on preliminary analyses. The task crafting sample items were: “Altered the scope or nature of work tasks to make better use of your personal strengths and skills” and “Altered the composition of work tasks (e.g., “by devoting extra time and effort to tasks you are passionate about”). The following examples reflected cognitive crafting: “tried thinking about negative aspects of your job in terms of challenges or learning opportunities” and “developed new ways of thinking about your work to make your job more meaningful and enjoyable”.

Individual growth-need strength was assessed using three items (α = 0.86) from the Job Diagnostic Survey [16] (e.g., “I would like to have opportunities to be creative and imaginative in my work.”). Intellectual stimulation was measured with a 3-item version (α = 0.95) of a core scale drawn from an established transformational leadership questionnaire [20], based on which respondents rated the behavior of their direct supervisor (e.g., “Has ideas that have challenged me to reexamine some of the basic assumptions about my work.”). To measure situational constraints, the three highest scoring items (α =0.86) from a longer organizational constraints scale were employed [33]. A sample item is: “too much ‘red tape’ frequently interferes with getting my work done”.

Psychological empowerment was assessed with an established validated scale [26,27], comprising three items for each of the four dimensions: (a) Self-determination (α = 0.80; e.g., “I have significant autonomy in determining how I do my job.”); (b) Impact (α = 0.87; e.g., “My impact on what happens in my department is large.”); (c) Meaning (α = 0.79; e.g., “The work I do is meaningful to me.”); and (d) Competence (α = 0.75; e.g., “I am confident about my ability to do my job.”).

A dichotomous item assessed gender (0 = male; 1 = female); age and organizational tenure (in years) were filled into designated number fields; education was measured with five categories.

### 2.3. Analyses

Confirmatory Factor Analysis (CFA) and Structural Equation Modeling (SEM) were conducted with AMOS 18.0, using conventional fit indices and cut-offs [34]: Incremental Fit Index (IFI), Tucker Lewis Index (TLI), and Comparative Fit Index (CFI) should at least be 0.90; Root Mean Square Error of Approximation (RMSEA) below 0.05 indicates close fit while up to 0.08 is acceptable; for the population RMSEA, a 90% confidence interval (CI) and a test whether its true value is higher than 0.05 are reported (should be non-significant); Hoelter’s Critical N (CN) is the theoretical sample size for which chi-square would not be significant (*p* > 0.05) and should be higher than 200.

## 3. Results

CFA models were tested in several steps (see Table 1). A two-factor model of task and cognitive job crafting (CFA1a) displayed good fit, whereas a single factor (CFA1b) was unacceptable. A three-factor model of growth and strength needs, intellectual stimulation, and organizational constraints (CFA2a) was suitable, but not a one-factor model (CFA2b). Corresponding with theory, psychological empowerment was adequately represented by four (CFA3a), but not by a single factor (CFA3b). Finally, all items were combined into a well-fitting nine-factor structure (CFA4a), whereas a one-factor model was rejected (CFA4b). The final CFA provided the basis for SEM. Directed paths were specified from antecedents on cognitive and task crafting, and from both forms of crafting on all four empowerment dimensions. As psychological empowerment is known to be influenced by individual differences, leadership, and work context [26,27], direct paths were added from all three antecedents on all four outcomes. Job crafting thus partially mediated these relationships. The structural model displayed close fit (SEM1a). Confirming Hypothesis 1, growth and strength needs related positively to both task (H1a: β = 0.32, *p* < 0.01) and cognitive crafting (H1b: β = 0.40, *p* < 0.01). Supporting Hypothesis 2, intellectual stimulation was positively associated with both task-directed (H2a: β = 0.11, *p* < 0.05) and cognitive approaches (H2b: β = 0.12, *p* < 0.01). Organizational constraints related positively only to task (H3a: β = 0.14, *p* < 0.01), but not to cognitive crafting (H3b: β = 0.06, *p* > 0.05), thus partially supporting Hypothesis 3. Differential effects on empowerment dimensions were confirmed. Task crafting related positively to self-determination (H4a: β = 0.14, *p* < 0.01) and impact (H4b: β = 0.18, *p* < 0.01), but not to meaning (β = −0.02, *p* > 0.05) or competence (β = −0.06, *p* > 0.05). Cognitive crafting showed an inverse pattern with positive effects on meaning (H4c: β = 0.22, *p* < 0.01) and competence (H4d: β = 0.32, *p* < 0.01), but not on self-determination (β = 0.05, *p* > 0.05) or impact (β = 0.03, *p* > 0.05). Additionally, growth need strength related to all empowerment dimensions (self-determination: β = 0.41, *p* < 0.01; impact: β = 0.41, *p* < 0.01; meaning: β = 0.41, *p* < 0.01; competence: β = 0.20, *p* < 0.01). Intellectual stimulation was associated with self-determination (β = 0.11, *p* < 0.01) and meaning (β = 0.17, *p* < 0.01), but not with impacts (β = −0.01, *p* > 0.05) or competence (β = −0.01, *p* > 0.05). Organizational constraints related negatively to self-determination (β = −0.07, *p* < 0.05) and meaning (β = −0.13, *p* < 0.01), but not to impact (β = 0.04, *p* > 0.05) or competence (β = −0.01, *p* > 0.05). In a further step, the control variables of gender, age, tenure, and education (SEM2b) were included. Model fit, effect sizes, and significance levels were stable. Tenure (β = 0.11, *p* < 0.01) and education (β = 0.07, *p* < 0.05) affected competence, while education was marginally related to self-determination (β = 0.06, *p* = 0.052) and impact (β = 0.05, *p* = 0.066). No other influences were found.

## 4. Discussion

This study is set apart from the majority of job crafting research because it does not draw on the reinterpretation of job crafting according to the job demands-resources model, but instead stays closer to the original and more open conceptualization. To address the lack of measures that reflect the original theorizing, we developed scales on cognitive and task crafting, which we integrated into a model of their antecedents and psychological consequences. Both the framing of job crafting as a form of self-empowerment and the contrast of situation-directed and self-directed strategies are unique to this study. In confirming dispositional influences, the need for growth and strength predicted both task-focused and mental crafting. The mixed previous results suggest that transformational leadership, specifically the intellectual stimulation of followers to think and act in new and different ways, facilitated both forms of crafting. Results on situational constraints only partially corresponded to expectations—as workers do not appear to use cognitive strategies to reframe such limiting conditions, but instead take actions to overcome them. Indeed, cognitive reframing may be ineffective in coping with situational constraints, reflecting hindrance stressors that pose obstacles to task fulfillment. However, for more ambiguous demands, such time pressure or an appraisal as either a threat or a challenge, may still be relevant.

The conceptualization of job crafting as self-empowerment was supported, as was the differential effects of situation-directed and self-directed strategies. Taken together, task and cognitive crafting explained additional variance in empowerment above and beyond growth need strength, intellectual stimulation, and situational constraints. The control-oriented dimensions of self-determination and impact were influenced by task-related crafting, that is, physical changes in the scope, number, or boundaries of job duties. Self-oriented dimensions of experienced meaning and competence, based on fit between purpose and requirements of the job and own values and skills, were positively affected by cognitive strategies of reframing one’s work role. Results support the relevance of job crafting as a “micro-emancipatory” form of self-management, through which workers expand opportunities for self-determination and influence, develop self-efficacy, and find meaning in their work. The quest for meaning at work appears to be a central driver in the motivation to engage in job crafting [29,35]. Future research should investigate how situation-directed and self-directed forms of task and cognitive job crafting interact in creating, maintaining, and increasing meaning at work. Depending on the context, enacting changes in the work situation and changes to oneself could reflect complementary, mutually reinforcing, alternative, or even compensatory strategies, e.g., cognitive reframing as a prerequisite, substitute, and/or consequence of active problem-solving. Framing job crafting more explicitly as a process of meaning-making and development of positive occupational identities, offers an opportunity to integrate and advance these different streams of research [35].

The rich psychological processes associated with job crafting hold the risk that research focuses too much on individual employees while neglecting their broader work environment. Job crafting does not occur in isolation or in a “vacuum”, but is embedded in social and organizational structures. For instance, managers can either frame and try to suppress task crafting as a form of disobedience or deviance, or recognize it as an active strategy that employees use to improve their intrinsic motivation and quality of working life. In fact, our results suggest that transformational leaders inspire workers to empower themselves by modifying their cognitive and task boundaries, thus facilitating positive work experiences [19,20,21]. As a micro-emancipatory behavior, job crafting is likely most beneficial for professional and personal development in work contexts by encouraging workers to strive to provide social support and respect for individual needs, preferences, and aspirations [3,8,35]. Exploring the positive roles job crafting can play in people-oriented management practices, thus, is a logical step. Updating notions of humanistic management, models of value-based, socially responsible, sustainable, and common good HR management have been suggested [36,37,38]. These models combine HR research with critical thinking on ecology and employment relationships, trying to reconcile, reduce, or balance tensions and trade-offs arising from structural conflicts of interests and power-dependence relationships. Incorporating somewhat similar assumptions, a link between job crafting and HR is the literature on idiosyncratic deals, i.e., personalized work and employment conditions, individually negotiated, and mutually beneficial to both employees and employer [39,40]. Work experiences are actualized at the intersection of “top-down” working conditions and “bottom-up” modifications and sense-making. Research should investigate interactions between job crafting and HR practices, including individual negotiation as a hybrid, bottom-up initiated and top-down authorized form of job personalization. From an integrative HR systems perspective, proactive job changes offer “secondary elasticities”, increasing responsiveness and flexibility in HR practices. Elaborating a focus on people-oriented HR, should not turn a blind eye on the stressful, detrimental, and impeding conditions many employees face in their workplaces on a daily basis, caused by high demands and performance pressure, lack of control and influence, injustice and insecurity [41]. Job crafting and idiosyncratic deals occur under both positive and negative conditions, though they are then likely to take on very different meanings.

Complementing the suggested focus on the positive roles of job crafting in employee-oriented HR systems, future research also needs to more directly address the limits and potential negative side-effects of job crafting, such as boundaries with counterproductive deviance. Although most empirical results support beneficial outcomes, job crafting may be psychologically effortful and alter intended work features. According to theory, job crafting can include a wide range of actions, e.g., expanding learning, reducing stressors, or helping others. Quantitative research, in particular, needs to be careful not to narrow down and restrict this broad conceptualization by selectively emphasizing some aspects over others. Job crafting is rooted in Western culture, emphasizing individualism, self-actualization, and constructive deviance. Our study demonstrates generalizability to the Chinese context, where initiative may challenge cultural values of collectivism, duty, and compliance. Although the overall prevalence of job crafting was high, self-directed changes were more common than situation-directed ones. Whether this finding is context-specific or generalizable requires further study. Limitations include cross-sectional self-report data; common method bias, however, is controversial and specified directions reflect common assumptions [42]. This does not exclude reciprocal relationships. It is likely that job crafting not only leads to empowerment, but also that empowered workers are prone to crafting their jobs to fit their actualization tendencies. Additional limitations involve ad hoc developed scales and exclusion of relational crafting for the sake of study parsimony. Controlling for growth need strength on empowerment shows that individual differences do matter, but do not offer an alternative explanation. As job crafting is dynamic, only longitudinal research designs can provide a deeper understanding of how people interact with their work over time.

## 5. Conclusions

Nowadays employees are increasingly recognized as co-designers of their jobs, using self-regulated actions to improve alignment between a person and their job by changing their tasks and themselves. Revisioning workers as active job crafters can enrich thinking on work design, leadership, coping, work motivation, and performance. A form of self-empowerment, job crafting is facilitated by individual and contextual factors. Enabling employees to apply effective and functional crafting strategies offers promising pathways to align, support, and enhance worker well-being, motivation, and performance.

## Figures and Tables

**Table 1:** Fit indices of confirmatory factor analyses and structural equation models.

Model	Description	χ^2^	df	IFI	TLI	CFI	RMSEA [CI]	CN
CFA1a	Job crafting (F = 2)	80.98 **	19	0.98	0.97	0.98	0.052 [0.041–0.064]	445
CFA1b	Job crafting (F = 1)	670.12 **	20	0.78	0.69	0.78	0.165 [0.154–0.176] **	57
CFA2a	Antecedents (F = 3)	85.98 **	24	0.99	0.99	0.99	0.046 [0.036–0.057]	507
CFA2b	Antecedents (F = 1)	2546.05 **	27	0.65	0.54	0.65	0.279 [0.270–0.189] **	19
CFA3a	Empowerment (F = 4)	215.52 **	48	0.97	0.96	0.97	0.054 [0.047–0.061]	362
CFA3b	Empowerment (F = 1)	2252.55 **	54	0.65	0.57	0.65	0.185 [0.178–0.191] **	39
CFA4a	All constructs (F = 9)	808.49 **	341	0.97	0.97	0.97	0.034 [0.031–0.037]	570
CFA4b	All constructs (F = 1)	9969.41 **	377	0.45	0.41	0.45	0.146 [0.143–0.148] **	51
SEM1a	Structural model	962.90 **	342	0.97	0.96	0.96	0.039 [0.036–0.042]	480
SEM1b	Controlled model	1166.32 **	422	0.96	0.95	0.96	0.038 [0.036–0.041]	483

Note: N = 1196; F = number of latent factors; χ^2^ = chi-square; df = degrees of freedom; IFI = Incremental Fit Index; TLI = Tucker Lewis Index; CFI = Comparative Fit Index; RMSEA = Root Mean Square Error of Approximation; CI = 90% Confidence Interval; CN = Hoelter’s Critical N; ** *p* < 0.01, * *p* < 0.05.
